# A novel near-infrared fluorescent probe for highly selective recognition of hydrogen sulfide and imaging in living cells†

**DOI:** 10.1039/c8ra03457e

**Published:** 2018-07-02

**Authors:** Keli Zhong, Longlong Deng, Jie Zhao, Xiaomei Yan, Tong Sun, Jianrong Li, Lijun Tang

**Affiliations:** College of Chemistry and Chemical Engineering, Bohai University Jinzhou 121013 China ljtang@bhu.edu.cn; College of Food Science and Technology, Bohai University, National & Local Joint Engineering Research Center of Storage, Processing and Safety Control Technology for Fresh Agricultural and Aquatic Products, The Fresh Food Storage and Processing Technology Research Institute of Liaoning Provincial Universities Jinzhou 121013 China jzsuntong@sina.com lijr6491@163.com; College of Laboratory Medicine, Dalian Medical University Dalian 116044 China

## Abstract

A novel near-infrared fluorescent probe (L) based on a 1,4-diethyl-1,2,3,4-tetrahydro-7*H*-pyrano[2,3-*g*]quinoxalin-7-one scaffold has been synthesized and characterized. Probe L displays highly selective and sensitive recognition to H_2_S over various anions and biological thiols with a large Stokes shift (125 nm) in THF/H_2_O (6/4, v/v, Tris–HCl 10 mM, pH = 7.4). This probe exhibits turn-on fluorescence for H_2_S through HS^−^ induced thiolysis of dinitrophenyl ether. Confocal laser scanning micrographs of MCF-7 cells incubated with L confirm that L is cell-permeable and can successfully detect H_2_S in living cells.

## Abstract 2

A novel “off–on” fluorescent probe (L) for H_2_S detection with NIR emission and imaging H_2_S in living cells.

## Graphical abstract

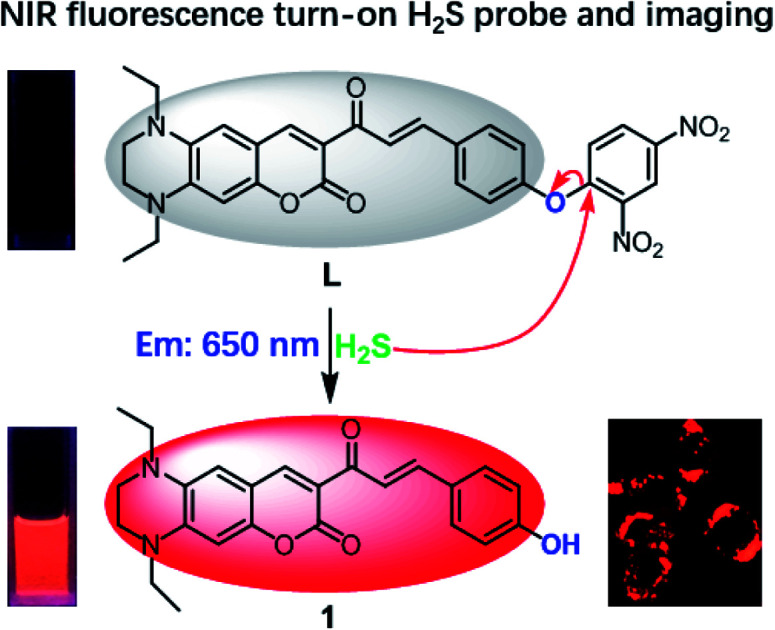

## Introduction

Hydrogen sulfide (H_2_S), a newly identified gaseous signalling molecule, has recently become a research focus in biological fields due to its multiple functions in physiological and pathological processes.^1^ Previous studies have demonstrated that endogenous H_2_S can affect the functions of neuronal, cardiovascular, immune, endocrine, and gastrointestinal systems and its antioxidant and anti-apoptotic signaling effects also show therapeutic benefit for the treatment of ischemia-induced heart failure.^2^ Excessive H_2_S can irritate the eyes and respiratory tract, causing severe loss of consciousness, respiratory failure, and even death.^3^ The concentration-dependent effects of H_2_S on health and disease demand precise methods to track the production of this important signaling molecule in living organisms.^4^

In view of the biological importance of H_2_S, several methods such as colorimetry,^5–8^ electrochemistry,^9–11^ and gas chromatography^12^ have been reported for H_2_S detection. Compared with these traditional methods, fluorescence analysis is widely used because of its advantages such as good selectivity, high sensitivity, simplicity, rapidness, and nondestructive bioimaging.^13^ Much attention has been given by chemists and biologists to develop various fluorescent chemosensors that would allow real-time tracking of a small molecule of interest in living cells and animals.^14^

In recent years, a number of fluorescent H_2_S probes based on different design strategies have been developed, including using nucleophilic substitution reaction,^15–17^ chemoselective reduction of azide to amine,^18–20^ Cu^2+^–fluorophore complex^21–23^ through indicator displacement assays. However, most of the reported H_2_S fluorescent probes still have some limitations, such as short emission wavelength, working in pure organic solvent, poor biological applications, *etc.* In fact, fluorescent probes with near-infrared (NIR) emission are more suitable for bioimaging applications due to their deep tissue penetration, minimum photo damage and background autofluorescence interference.^24–32^ Therefore, it is important to design and synthesize NIR fluorescent probe for selective and sensitive detection of H_2_S.^33–35^

In view of this, we judiciously designed an “off–on” fluorescence probe (L) for H_2_S detection with NIR emission. Coumarinyl chalcone (1) was selected as the NIR emitting fluorophore owing to its high molar absorption coefficient and long-wavelength emission.^36^ The incorporated 2,4-dinitrophenyl ether moiety in L was anticipated to function as both H_2_S recognition site and fluorescence quencher.^37–41^ After reaction with H_2_S, a fluorescence turn-on response would occur through HS^−^-triggered thiolysis of dinitrophenyl ether to release 1. Further investigations demonstrate that the proposed fluorescent probe L displays high selectivity and sensitivity to H_2_S with fast response and NIR emission, and L is applicable to image H_2_S in living cells. To the best of our knowledge, NIR fluorescence turn-on H_2_S probe based on thiolysis of the dinitrophenyl ether were rarely reported.

## Experimental

### Instruments and materials

^1^H NMR and ^13^C NMR spectra were measured on an Agilent 400 MR spectrometer, and the chemical shifts were expressed in ppm and coupling constants (*J*) in hertz. High-resolution mass spectra (HRMS) were measured using a Bruker micrOTOF-Q mass spectrometer (Bruker Daltonik, Bremen, Germany). Fluorescence measurements were carried out on a Sancho 970-CRT spectrofluorometer (Shanghai, China). UV-vis absorption spectra were measured with a SP-1900 spectrophotometer (Shanghai Spectrum Instruments Co., Ltd., China). pH measurements were made with a Model PHS-25 Bmeter (Shanghai, China). Cell imaging was observed under a confocal laser scanning microscope (LEICA TCS SP5 II, Germany) with excitation at 405 nm. Fluorescence quantum yields were measured with an absolute fluorescence quantum yield spectrometer (Quantaurus-QY C11347, Hamamatsu Photonics).

Unless otherwise noted, reagents were purchased from commercial suppliers and used without further purification. 3 was prepared according to the reported method^42^ (ESI†). MCF-7 (human breast carcinoma) cells were obtained from Institute of Basic Medical Sciences (IBMS) of Chinese Academy of Medical Sciences (CAMS).

### General methods

Stock solutions (50 mM) of anions (sodium or potassium salts), NaHS used as H_2_S source, and biological thiols (glutathione (GSH), homocysteine (Hcy) and cysteine (Cys)) were prepared in double-distilled water. Stock solution of L (10 mM) was prepared in DMSO and was further diluted with a mixed solution of THF/H_2_O (v/v, 6 : 4 Tris–HCl 10 mM, pH = 7.4) to make a final concentration at 10 μM. Fluorescence spectra were measured using 10 μM solution of L after 30 minutes upon addition of anions by excitation at 500 nm. The excitation and emission slit widths were 10 and 10 nm, respectively. All titration experiments were carried out at room temperature. Double-distilled water was used throughout the experiments.

### Synthesis of compound 2

Compound 3 (468.2 mg, 2.0 mmol), acetylacetic ether (260.4 mg, 2.0 mmol), and piperidine (0.1 mL) were dissolved in dry ethanol, the mixture was heated to refluxed for 4 h, then it was poured into hydrochloric acid (4 M, 10 mL) and stirred at 60 °C for 30 minutes. After that, the mixture was further diluted with H_2_O and extracted with ethyl acetate, and organic layer was dried with anhydrous Na_2_SO_4_ and removed under reduced pressure. The residue was purified by column chromatography with petroleum ether/ethyl acetate (5 : 1, v/v) as eluent to give compound 2 (420.5 mg, 70%). ^1^H NMR (400 MHz, CDCl_3_) *δ* 8.38 (s, 1H), 6.48 (s, 1H), 6.39 (s, 1H), 3.59–3.52 (m, 2H), 3.42 (q, *J* = 7.1 Hz, 2H), 3.32 (q, *J* = 7.1 Hz, 2H), 3.26–3.20 (m, 2H), 2.67 (s, 3H), 1.22 (t, *J* = 7.2 Hz, 3H), 1.18 (t, *J* = 7.2 Hz, 3H).

### Synthesis of compound 1

Compound 2 (300.4 mg, 1.0 mmol), *p*-hydroxybenzaldehyde (134.2 mg, 1.1 mmol), and piperidine (0.1 mL) were dissolved by toluene, the mixture was heated to refluxed for 4 h, then it was poured into hydrochloric acid (4 M, 10 mL) and stirred for 50 min at 60 °C. After that, water were added into the mixture, then mixture was extracted with ethyl acetate (3 × 100 mL), and combined organic layer was dried with anhydrous Na_2_SO_4_ and removed under reduced pressure. The crude products were purified by column chromatography with petroleum ether/ethyl acetate (1 : 1, v/v) as eluent to give compound 1 (199.8 mg, 49%). Mp 203.2–203.7 °C; ^1^H NMR (400 MHz, DMSO-*d*_6_) *δ* 10.01 (s, 1H), 8.48 (s, 1H), 7.82 (d, *J* = 15.7 Hz, 1H), 7.56 (d, *J* = 15.7 Hz, 1H), 7.51 (d, *J* = 8.2 Hz, 2H), 6.76–6.83 (m, 3H), 6.51 (s, 1H), 3.51 (t, *J* = 5.1 Hz, 2H), 3.45 (q, *J* = 7.1 Hz, 2H), 3.27 (d, *J* = 7.1 Hz, 2H), 3.15 (t, *J* = 5.1 Hz, 2H), 1.04–1.12 (m, 6H); ^13^C NMR (101 MHz, DMSO-*d*_6_) *δ* 185.70, 160.26, 153.36, 147.86, 143.95, 142.54, 132.69, 131.95, 130.80, 130.06, 129.09, 126.50, 122.21, 116.36, 115.25, 109.00, 108.14, 95.06, 47.53, 45.96, 45.05, 44.11, 10.85, 9.64; HRMS (ESI^+^) calcd for C_24_H_24_N_2_O_4_ [M − H]^+^: 403.1736, found: 403.1658.

### Synthesis of probe L

Compound 1 (404.5 mg, 0.5 mmol), 2,4-dinitrofluorobenzene (186.0 mg, 0.6 mmol), potassium carbonate (187 mg, 1.0 mmol) were dissolved into DMF (5.0 mL), the mixture was stirred for 3 h at room temperature. After that, water was added and then the mixture was extracted with ethyl acetate, organic layer was dried with anhydrous Na_2_SO_4_. The crude products was purified by column chromatography with petroleum ether/dichloromethane (1 : 4, v/v) as eluent to give compound L (285.3 mg, 50%). Mp > 250 °C; ^1^H NMR (400 MHz, DMSO-*d*_6_) *δ* 8.91 (d, *J* = 2.8 Hz, 1H), 8.55 (s, 1H), 8.46 (dd, *J* = 9.2, 2.8 Hz, 1H), 8.04 (d, *J* = 15.8 Hz, 1H), 7.87 (d, *J* = 8.4 Hz, 2H), 7.70 (d, *J* = 15.8 Hz, 1H), 7.35 (d, *J* = 8.4 Hz, 2H), 7.30 (d, *J* = 9.2 Hz, 1H), 6.88 (s, 1H), 6.57 (s, 1H), 3.57 (t, *J* = 5.0 Hz, 3H), 3.50 (q, *J* = 7.0 Hz, 2H), 3.36–3.31 (m, 2H), 3.20 (t, *J* = 5.0 Hz, 2H), 1.18–1.09 (m, 6H); ^13^C NMR (101 MHz, DMSO-*d*_6_) *δ* 185.76, 160.79, 155.52, 154.78, 153.64, 148.07, 144.29, 142.17, 140.44, 140.04, 133.47, 132.76, 131.96, 131.10, 130.11, 129.09, 126.24, 122.37, 121.08, 120.38, 114.76, 109.10, 108.14, 95.08, 47.59, 46.03, 45.06, 44.06, 10.89, 9.63; HRMS (ESI^+^) calcd for C_30_H_27_N_4_O_8_ [M + H]^+^: 571.1751, found: 571.1802.

### Cell viability assays

The cytotoxicity of the probe was examined by MTT assay. MCF-7 cells with 90% confluence was digested by 0.25% of trypsin, and transferred into 96-well plates with a cell suspension of 5000 cells per hole. The cells were incubated for 24 h at 37 °C, and then different concentrations of L (0, 1.0, 5.0, 10, 20, 40 μM) were added to the 96-well plates. After 24 h incubation at the same condition, 10 μL MTT was added and incubated for another 4 h. The cells were washed twice with PBS (1 mL) and dissolved with DMSO (1 mL). The absorbance was recorded at 490 nm using the microplate spectrophotometer system, and each experiment was run in triplicate. Cell viability was calculated based on the equation: Cell viability (%) = (*A*_with probe_ − *A*_blank_)/(*A*_control_ − *A*_blank_) × 100%.

### Cell culture and confocal image

Similar to the cytotoxicity assay and incubation conditions, MCF-7 cells of proper density were transferred in confocal dishes. After 24 h incubating at 37 °C, the cell layer were washed three times with PBS buffer, then the same solution with a concentration of 2 μM L was added into each well and incubated for 30 min. Subsequently, the L treated MCF-7 cells were washed three times with PBS, and then further incubated with different concentrations of NaHS (10, 20, and 50 μM) for 30 min. After washing the culture dishes three times with PBS, fluorescence imaging experiments were performed using a LEICA TCS SP5 II confocal laser scanning microscope.

## Results and discussion

### Synthesis and characterization of probe L

**Scheme 1:**
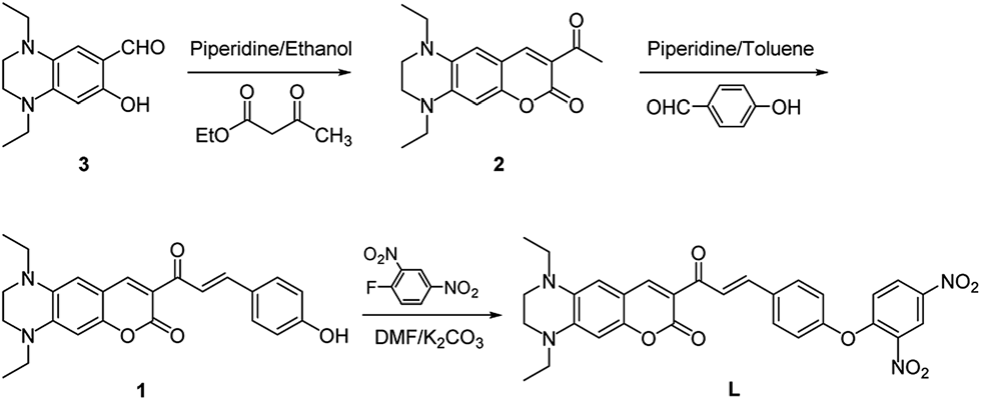
Synthetic route to probe L.

Probe L was synthesized according to the procedures as outlined in Scheme 1. Condensation of 1,4-diethyl-7-hydroxy-1,2,3,4-tetrahydroquinoxaline-6-carbaldehyde (3) with ethyl acetoacetate to give compound 2, which was followed by Claisen–Schmidt reaction with *p*-hydroxy benzaldehyde to afford compound 1. Probe L was synthesized *via* nucleophilic substitution reaction of 1 with 2,4-dinitrofluorobenzene and was structurally characterized by ^1^H NMR, ^13^C NMR, HRMS (Fig. S1–S3, ESI†).

### Optical responses of L to H_2_S

**Fig. 1:**
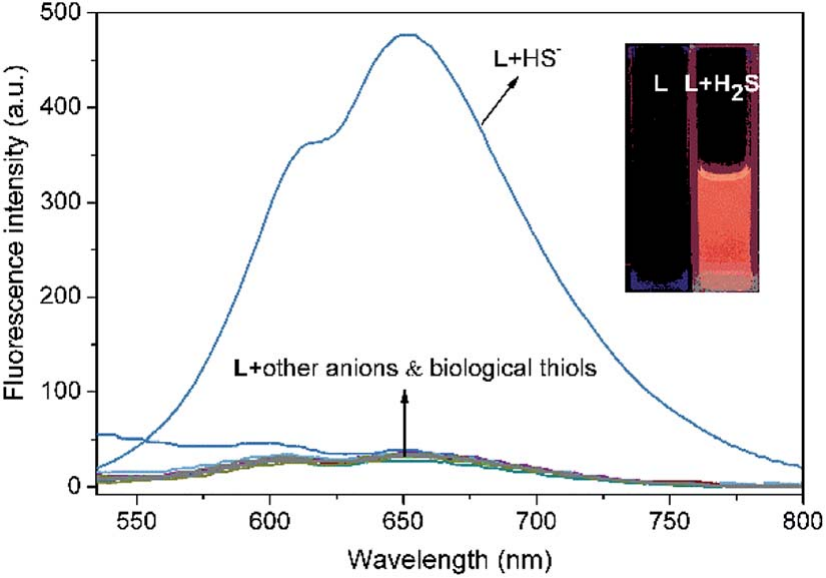
Fluorescence spectra of L (10 μM) upon addition of F^−^, Cl^−^, Br^−^, I^−^, NO_2_^−^, CO_3_^2−^, HCO_3_^−^, CH_3_COO^−^, HPO_4_^2−^, H_2_PO_4_^−^, PO_4_^3−^, CN^−^, SCN^−^, HS^−^, PPi, ClO^−^, SO_4_^2−^, SO_3_^2−^, HSO_3_^−^, HSO_4_^−^, N_3_^−^ and S_2_O_3_^2−^ and biological thiols (Cys, Hcy, GSH) in THF–Tris (6/4, v/v) solution (*λ*_ex_ = 500 nm).

The specific selectivity of a probe determines its basic performance. Therefore, the optical responses of L toward various anions including F^−^, Cl^−^, Br^−^, I^−^, NO_2_^−^, CO_3_^2−^, HCO_3_^−^, CH_3_COO^−^, HPO_4_^2−^, H_2_PO_4_^−^, PO_4_^3−^, CN^−^, SCN^−^, HS^−^, PPi, ClO^−^, SO_4_^2−^, SO_3_^2−^, HSO_3_^−^, HSO_4_^−^, N_3_^−^ and S_2_O_3_^2−^, as well as biological thiols (Cys, Hcy, GSH) were explored (Fig. 1). Probe L (10 μM) displays quite weak fluorescence emission (*Φ* = 0.0035) in THF/H_2_O (6/4, v/v, Tris–HCl 10 mM, pH = 7.4) solution (*λ*_ex_ = 500 nm). Due to the strong electron withdrawing effect of the 2,4-dinitrophenyl ether group, a possible donor-excited photo-induced electron transfer (d-PET) process from coumarinyl chalcone to 2,4-dinitrophenyl would occur on excitation,^43^ which is responsible to the observed fluorescence quenching. When L was treated with 100 equiv. of HS^−^, a strong emission band centered at 650 nm occurred (*Φ* = 0.011) and the fluorescence color changed from non-fluorescence to vivid red (Fig. 1, inset or S4†). However, the fluorescence emission intensity of L underwent negligible or very slight changes upon addition of other anions and biological thiols to solution of L. These results demonstrate that L possesses high selectivity to H_2_S.

**Fig. 2:**
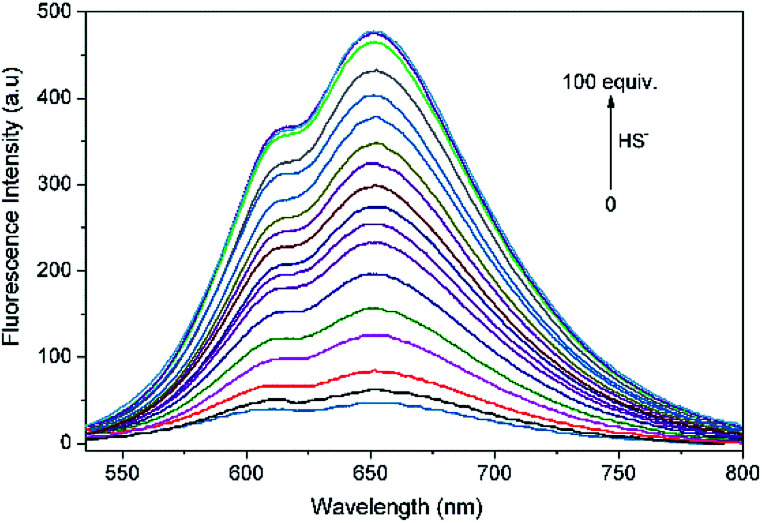
Fluorescence spectrum changes of L (10 μM) upon incremental addition of HS^−^ (0–100 equiv.) in THF–Tris (6/4, v/v) solution (*λ*_ex_ = 500 nm).

In addition, UV-vis absorption experiments were carried out, and the absorption band centered at 525 nm of L (*ε* = 428 144 M^−1^ cm^−1^) underwent a blue shift of 8 nm upon addition of HS^−^ (*ε* = 391 138 M^−1^ cm^−1^). There was no significant change in the absorption and color of L solution upon addition of other anions and biological thiols (Fig. S5†). The results indicate that L is hardly to recognize H_2_S *via* UV-vis measurements. The Stokes shift was found to be 125 nm, such a large Stokes shift and emission wavelength are beneficial to biological imaging because they could efficiently minimize self-absorption and reduce the interference from auto-fluorescence (Fig. 2).^44^

Fluorescence titration experiments of L with HS^−^ were performed to further explore the sensing behaviors of L. With increasing the added amount of HS^−^ to L solution, the emission intensity of L at 650 nm concomitantly increased and reached a plateau when 100 equiv. of HS^−^ was employed. The intensity of L towards HS^−^ exhibited a good linear correlation with the concentration of HS^−^ ranging from 200 to 550 μM (*R*^2^ = 0.9938). Based on the signal to noise ratio, the detection limit (LOD = 3*σ*/*k*, *σ* is the standard deviation of the blank solution; *k* is the slope of the calibration curve) of L to HS^−^ was calculated to be 7.3 × 10^−7^ M (Fig. S6†), indicating that L was highly sensitive to H_2_S and had a potential applicability for bioimaging of HS^−^.

**Fig. 3:**
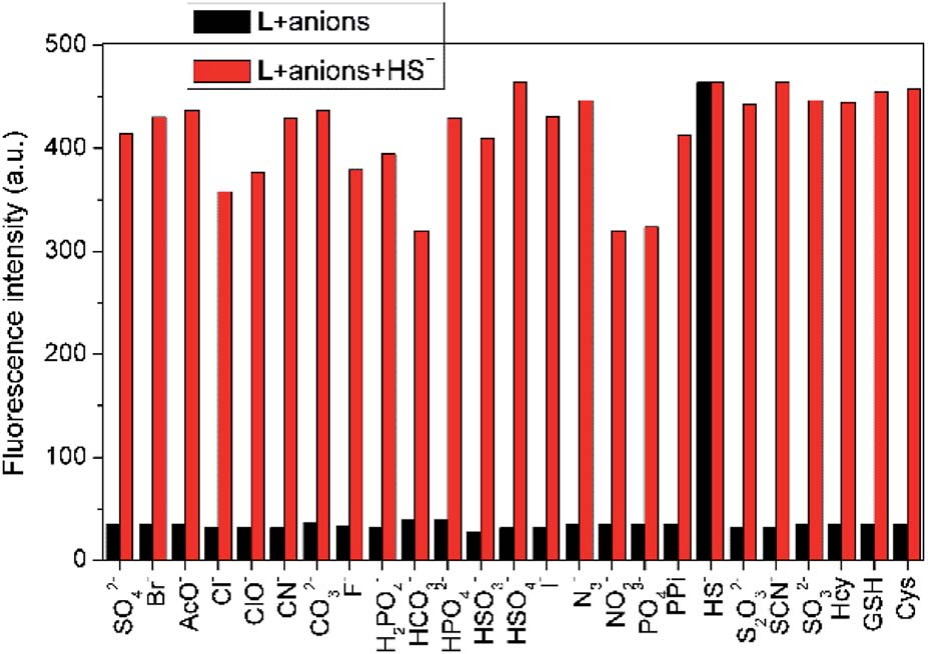
Fluorescence intensity (*λ*_em_ = 650 nm) changes of L (10 μM) in THF–Tris (6/4, v/v) solution upon sequential addition of various anions and biological thiols (100 equiv.) and HS^−^ (100 equiv.).

**Fig. 4:**
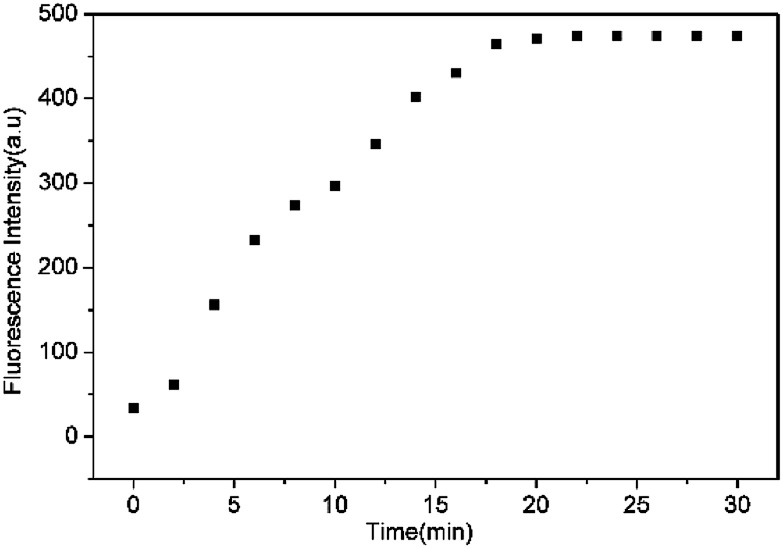
Plot of the emission intensity at 650 nm of L (10 μM) upon addition of HS^−^ (100 equiv.) in THF–Tris (6/4, v/v) solution against with time.

To assess the specific nature of L toward HS^−^, competitive experiments were then performed to estimate the availability of L in complicated systems. As illustrated in Fig. 3, HS^−^ could still produce a significant fluorescence enhancement in the presence of co-existing anions or biothiols, indicating the excellent anti-interference ability of L for HS^−^ recognition. At the same time, we also prove that S_2_^2−^ and *p*-toluenethiol have no interference for recognition of H_2_S (Fig. S7†), which is beneficial to its potential applications in complicated biological systems. In addition, the time-dependent fluorescence variations of the probe were also monitored. As shown in Fig. 4, the fluorescence intensity of L increased along with the time and reached a maximum within ∼18 min, which is faster than that of some reported probes (Table S1†), indicative of a fast response of L for H_2_S detection.

**Fig. 5:**
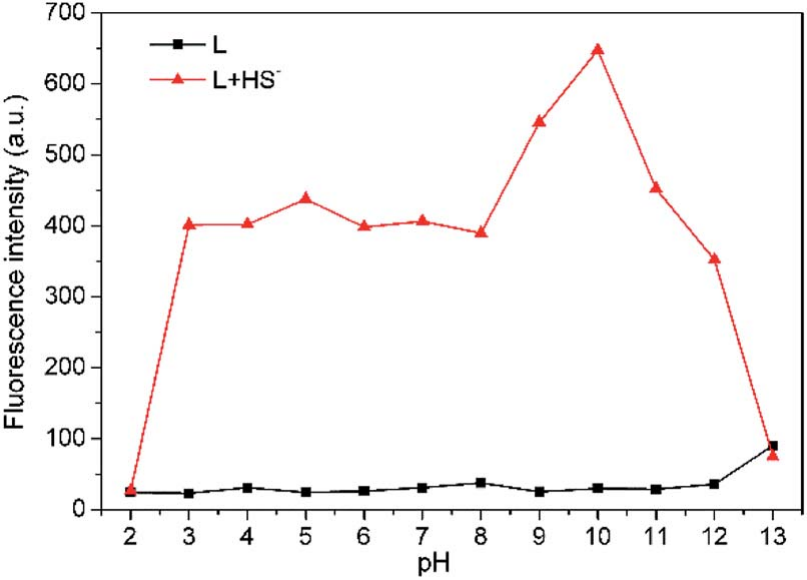
Effects of pH on fluorescence intensity at 650 nm of L (10 μM) in THF–Tris (6/4, v/v) solution with (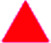) or without (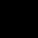) HS^−^.

For biological applications, pH dependence of L was examined in THF–Tris (6/4, v/v) solution (Fig. 5). Probe L is pH insensitive in a wide pH range from 2 to 12. However, there is significant fluorescence enhancement with addition of HS^−^ when L is within pH range from 3 to 12, which includes the biologically relevant range of pH 4–8, indicating that L is applicable to detect H_2_S in the biological system.

### The sensing mechanism of L to H_2_S

**Scheme 2:**
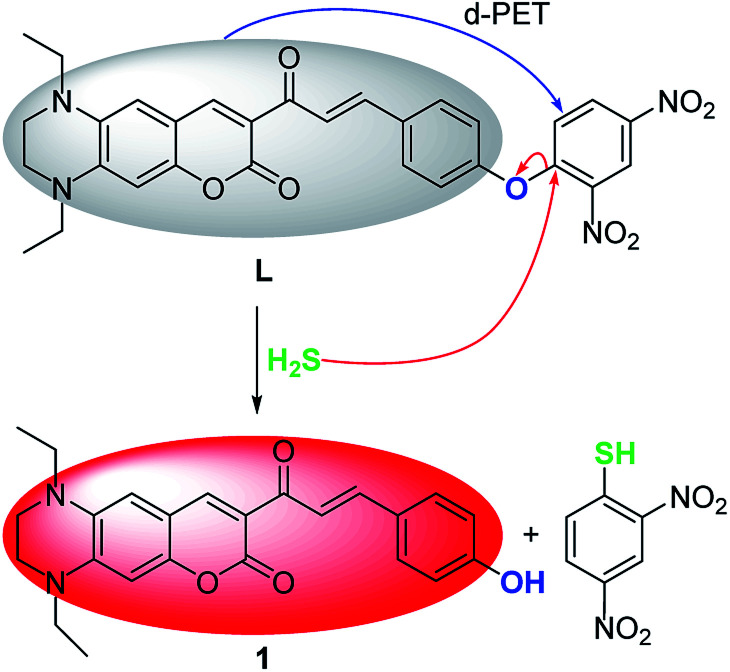
The sensing mechanism of L for H_2_S.

Preliminary investigations reveal that the fluorescence spectrum of L + HS^−^ exhibits an almost identical emission pattern as that of free 1 (Fig. S8†), suggesting that HS^−^ can completely cleave the dinitrophenyl ether moiety to release 1.^45–47^ To further corroborate this reaction process, HRMS of the reaction mixture L + HS^−^ was analyzed (Fig. S9†). The prominent peak observed at *m*/*z* = 405.1816 can be ascribed to reaction released compound 1 (Calcd *m*/*z* = 405.1815). These results reveal that HS^−^-triggered thiolysis of dinitrophenyl ether to release fluorescent dye 1 indeed happened. The sensing mechanism of L toward H_2_S was proposed in Scheme 2.

### Live cell imaging of L to H_2_S

**Fig. 6:**
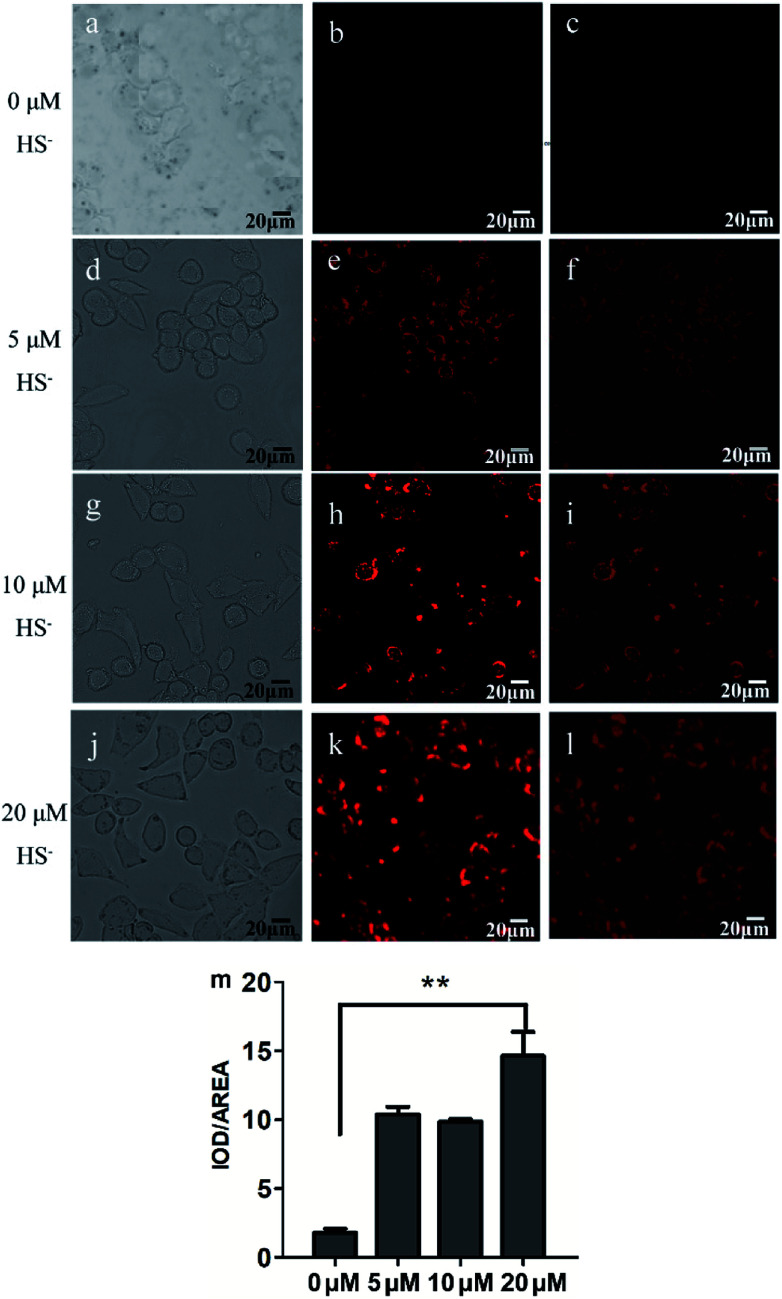
Bright-field (a, d, g and j), dark field (b, e, h and k), overlay images (c, f, i and l) of MCF-7 cells after successive incubation with L (2 μM) and different concentrations of HS^−^ for 30 min; the integrated optical density analysis from dark field (m), **P > 0.01.

To validate the biological applicability of L, live cell imaging experiments were also performed using MCF-7 cells. Firstly, the cytotoxicity of the probe was evaluated by MTT assay (Fig. S10†). The results showed that the cell viability was estimated to be >88% at 24 h when L was used less than 10 μM. Thus 2 μM of the probe was selected as non-cytotoxic dose for further studies. Subsequently, MCF-7 cells were incubated with L (2 μM) for 30 min at 37 °C and then washed three times with PBS buffer, there was negligible fluorescence signal in the red channel (Fig. 6b). When L-pretreated MCF-7 cells were further incubated with different concentrations of H_2_S (5, 10, and 20 μM) for 30 min, the brightness of the observed red fluorescence from the red channel gradually increased with increase of H_2_S concentration (Fig. 6e, h and k). The integrated optical density analysis manifests that the fluorescence intensity was significantly increased after addition 20 μM of HS^−^, which has prominent statistically significance compared with the control group (*P* > 0.01, Fig. 6m). These results indicate that L possesses good cell permeability and is capable of imaging HS^−^ in MCF-7 cells.

## Conclusion

In summary, we reported herein a unique NIR fluorescent turn-on H_2_S probe based on thiolysis of dinitrophenyl ether. Probe L displays highly selective and sensitive recognition to H_2_S over various anions and biological thiols with a large Stokes shift in THF/H_2_O (6/4, v/v, Tris–HCl 10 mM, pH = 7.4) solution. Moreover, probe L is suitable for fluorescence imaging of H_2_S in living MCF-7 cells. Based on the unique fluorescence feature, the coumarinyl chalcone conjugate (dye 1) will be a promising platform for the development of various NIR fluorescent probes.

## Conflicts of interest

There are no conflicts to declare.

## Supplementary Material

RA-008-C8RA03457E-s001
