# Supplementary material for: A novel near-infrared fluorescent probe for highly selective recognition of hydrogen sulfide and imaging in living cells
Source: RSC Adv. 2018 Jul 2;8(42):23924–9. doi: 10.1039/c8ra03457e (PMC9081853; doi:10.1039/c8ra03457e)
Supplement: RA-008-C8RA03457E-s001 [file RA-008-C8RA03457E-s001.pdf]

Electronic Supplementary Material (ESI) for RSC Advances.
This journal is © The Royal Society of Chemistry 2018

Electronic Supplementary Information

A novel near-infrared fluorescent probe for highly selective recognition of hydrogen sulfide and imaging in living cells

Keli Zhong[a, b], Longlong Deng,[a] Jie Zhao[a], , Xiaomei Yan[c], Tong Sun* [b], Jianrong Li* [b], and Lijun Tang* [a]

[a] *College of Chemistry and Chemical Engineering, Bohai University, Jinzhou, 121013, China*

[b] *College of Food Science and Technology, Bohai University; National & Local Joint Engineering Research Center of Storage, Processing and Safety Control Technology for Fresh Agricultural and Aquatic Products; The Fresh Food Storage and Processing Technology Research Institute of Liaoning Provincial Universities, Jinzhou, 121013, China*

[c] *College of Laboratory Medicine, Dalian Medical University, Dalian, 116044, China*

* *Corresponding author. Fax: 86-416-3400158; Tel: 86-416-3400302;*

E-mail: jzsuntong@sina.com (T. Sun), lijr6491@163.com (J. Li) and ljtang@bhu.edu.cn (L. Tang)

Contents

Page No.

Figure S1. ^{1}H NMR spectrum of compound **L** in DMSO-d_6.............................S3

Figure S2. ^{13}C NMR spectrum of compound **L** in DMSO-d_6............................S3

Figure S3. HRMS spectrum of compound **L** in CH_3CN..............................S4

Figure S4. Fluorescence changes of various anions added to probe **L**.................S4

Figure S5. UV–vis absorption of various anions added to **L** S4

Figure S6. The detection limit of probe **L** to HS^- S5

Figure S7. The selectivity and anti-interference of **L** for $S_2^{2-}$and *p*-toluenethiol......S5

Figure S8. Fluorescence spectra of **L**, **L**+HS^- and compound **1**...................... S5

Table S1. Comparison of **L** with other reported fluorescence probes....................S6

Figure S9. HRMS of **1** and the reaction mixture of **L** with **HS**$^-$.......................S7

Figure S10. Cell viability values estimated by MTT assay in MCF-7 cells...........S7

Scheme S1. Synthesis route of **L**..S8

Figure S11. ^{1}H NMR spectrum of compound **6** in $CDCl_3$..............................S9

Figure S12. ^{1}H NMR spectrum of compound **5** in $CDCl_3$..............................S10

Figure S13. ^{1}H NMR spectrum of compound **4** in $CDCl_3$..............................S11

Figure S14. ^{1}H NMR spectrum of compound **3** in DMSO-d_6.........................S12

Figure S15. ^{1}H NMR spectrum of compound **2** in $CDCl_3$..............................S13

Figure S16. ^{1}H NMR spectrum of compound **1** in DMSO-d_6.........................S13

Figure S17. ^{13}C NMR spectrum of compound **1** in DMSO-d_6.......................S14

Figure S18. HRMS spectrum of compound **1** in CH_3CN……………………….………S14

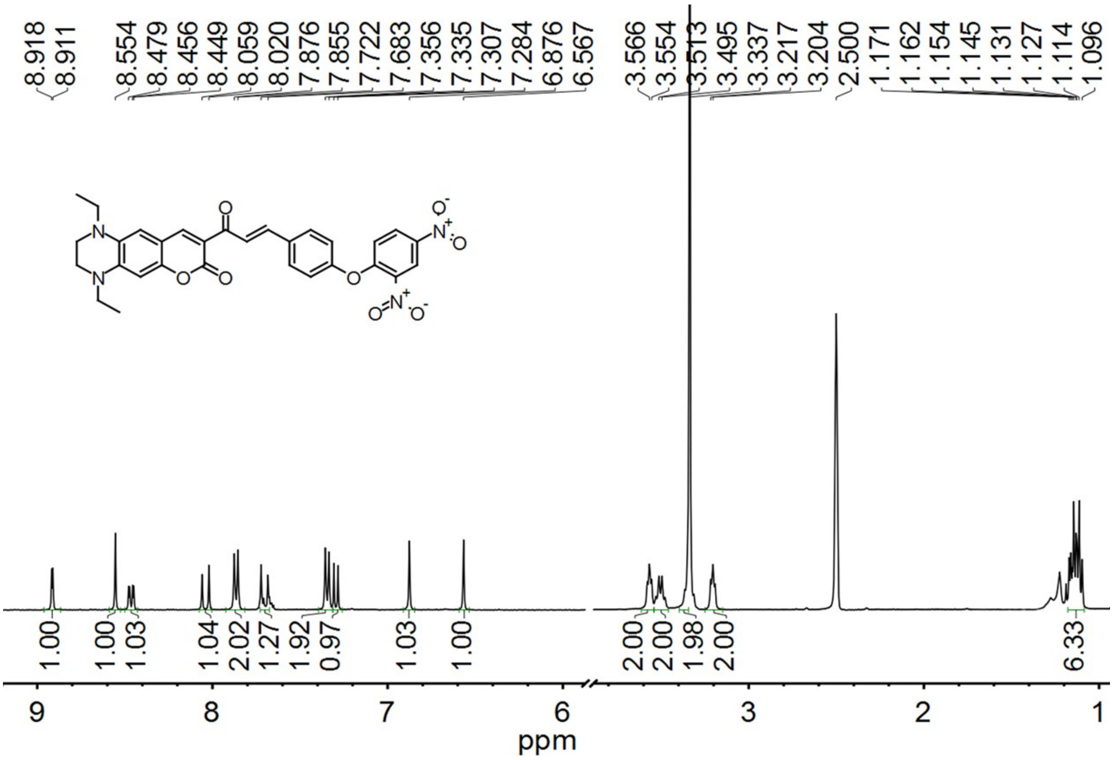

Figure S1. ^{1}H NMR spectrum of compound **L** in DMSO-d_6.

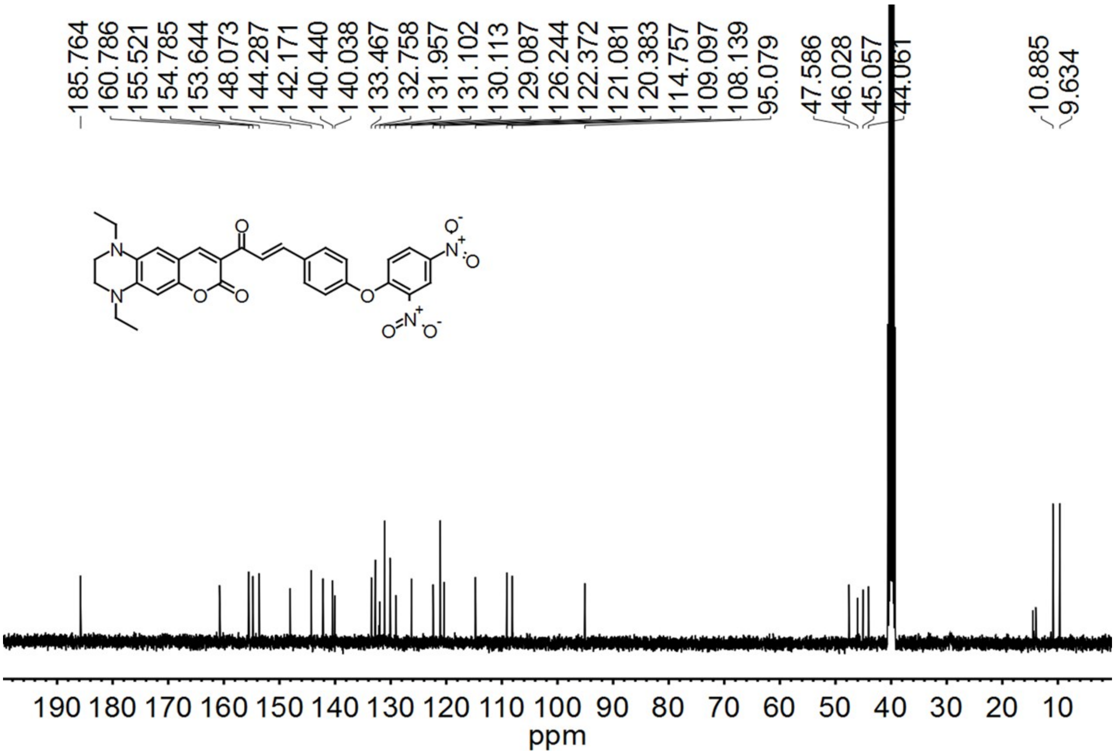

Figure S2. ^{13}C NMR spectrum of compound **L** in DMSO-d_6.

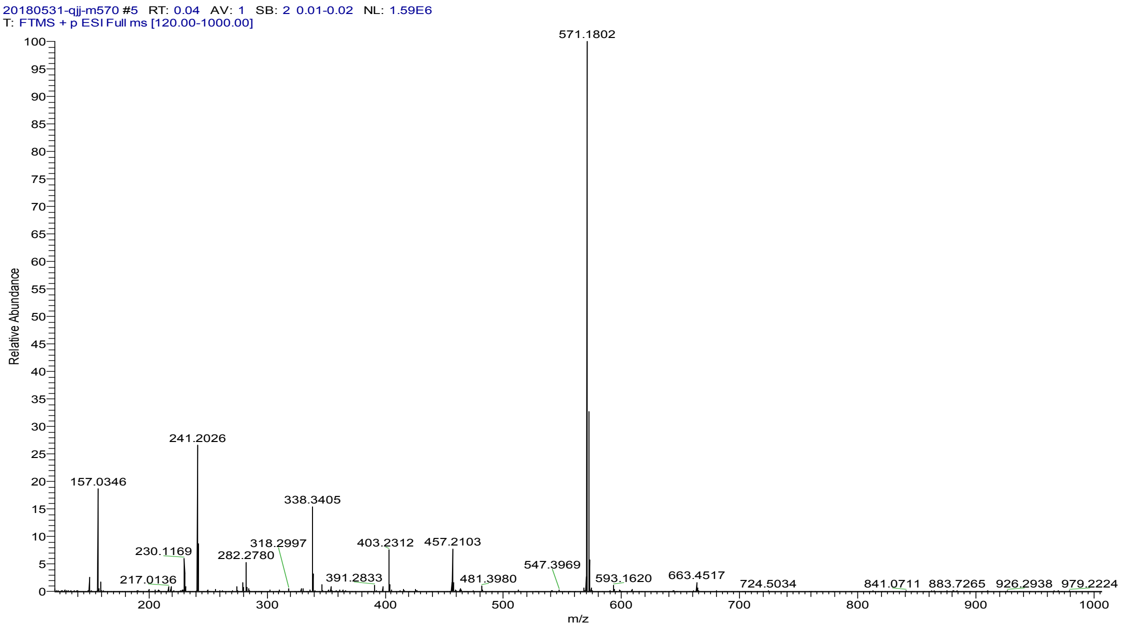

Figure S3. HRMS spectrum of compound **L** in CH_3CN ($[M+H]^+$ calcd: 571.1751, found: 571.1802) .

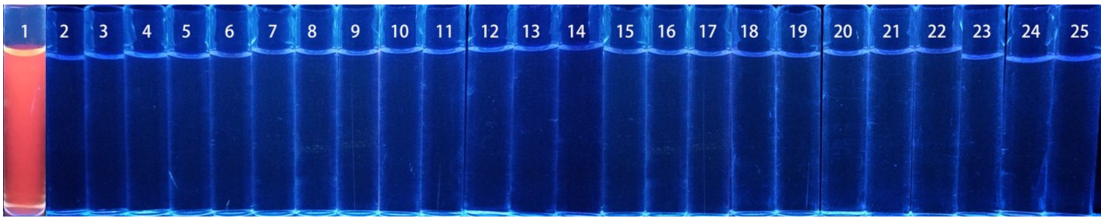

Figure S4. Fluorescence changes of various anions added to probe **L**, 1: HS^-, 2: F^-, 3: Cl^-, 4: Br^-, 5: I^-, 6: NO_2^-, 7: CO_3^{2-}, 8: HCO_3^-, 9: CH_3COO^-, 10: HPO_4^{2-}, 11: $H_2PO_4^-$, 12: PO_4^{3-}, 13: CN^-, 14: SCN^-, 15: PPi, 16: ClO^-, 17: SO_4^{2-}, 18: SO_3^{2-}, 19: HSO_3^-, 20: HSO_4^-, 21: N_3^-, 22: $S_2O_3^{2-}$, 23: Hcy, 24: GSH, 25: Cys.

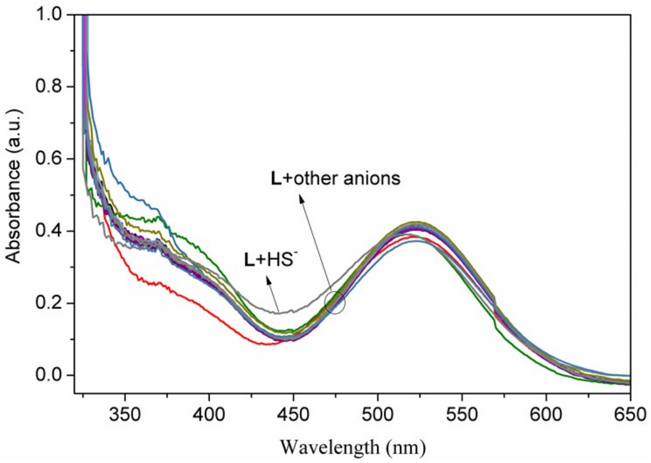

Figure S5. UV–vis absorption spectra of probe **L** on addition of various anions in THF/Tris (6/4, v/v, pH = 7.4) solution.

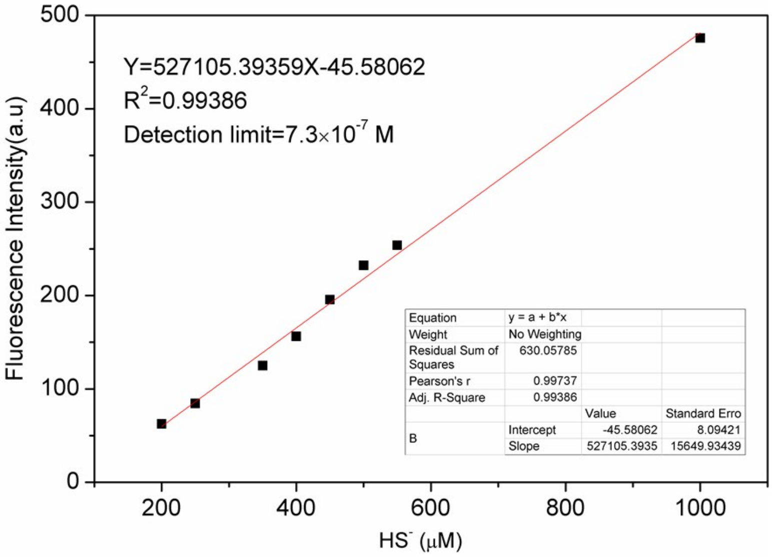

Figure S6. The detection limit of probe **L** to HS^-.

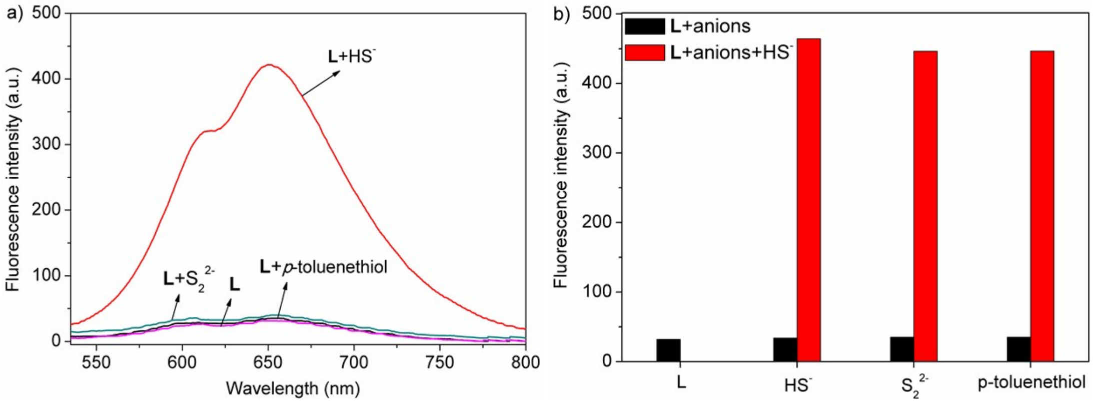

Figure S7. The selectivity a) and anti-interference b) of **L** for $S_2^{2-}$and *p*-toluenethiol.

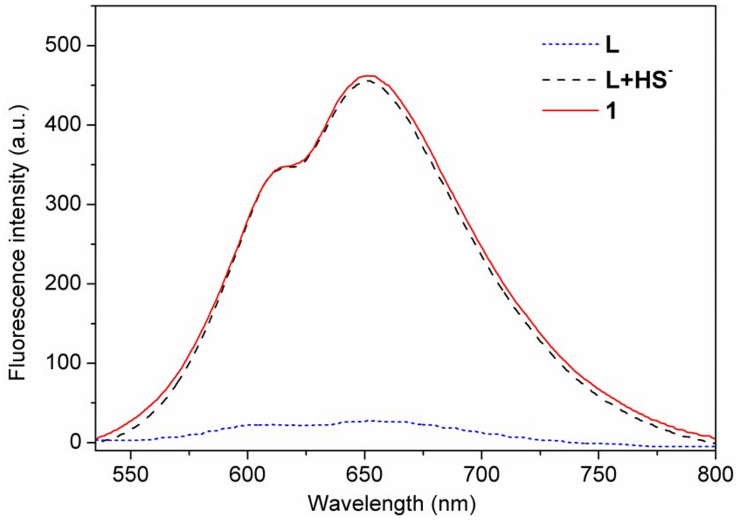

Figure S8. Fluorescence spectra of **L**, **L**+HS^- and compound **1**.

Table S1. Comparison of the proposed probe with other reported fluorescence probes for the detection of H_2S.

No.	Molecular structure	λ_{ex} /nm	λ_{em} /nm	Stokes shift /nm	Response time/min	Literature
1	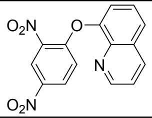	450	514	~64	50	RSC Adv., 2013, 3, 25690-25693
2	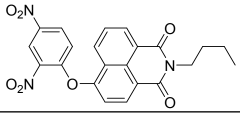	450	550	~100	20	Dyes Pigm., 2013, 99, 537-542
3	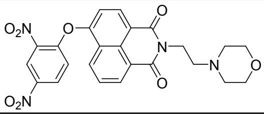	450	550	105	20	Org. Lett., 2013, 15, 2310-2313
4	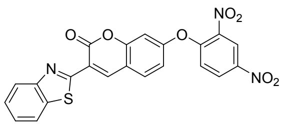	460	497	37	25	Sens. Actuators, B, 2014, 196, 151-155
5	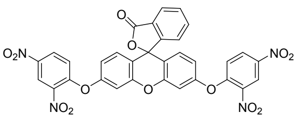	450	525	75	40	Chin. Chem. Lett., 2014, 25, 1060-1064
6	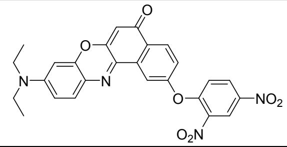	530	655	125	20	Sens. Actuators, B, 2014, 202: 99-104
7	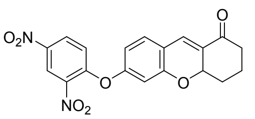	410	514	104	40	Anal. Chim. Acta, 2015, 853, 548-554
8	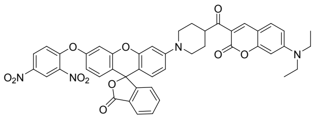	418	541	~123	30	Dyes Pigm., 2015, 118, 88-94
9	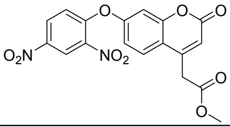	318	463	78	60	Anal.Methods, 2015, 7, 7646-7652
10	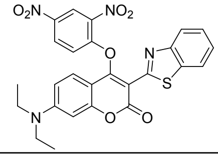	370	424	~54	150	Sens. Actuators, B, 2016, 232, 705-711
11	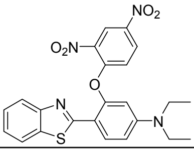	360	504	~144	30	Anal.Methods, 2016, 8, 6832-6839
12	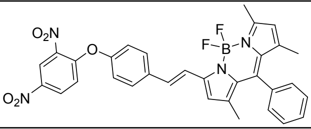	574	592	18	20	Talanta, 2018, 181, 104-111
13	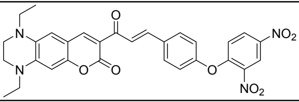	500	650	125	18	This work

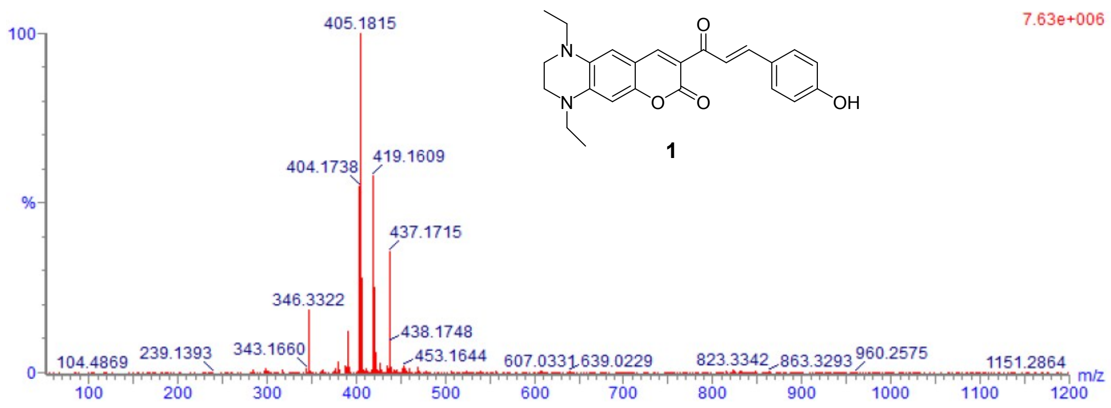

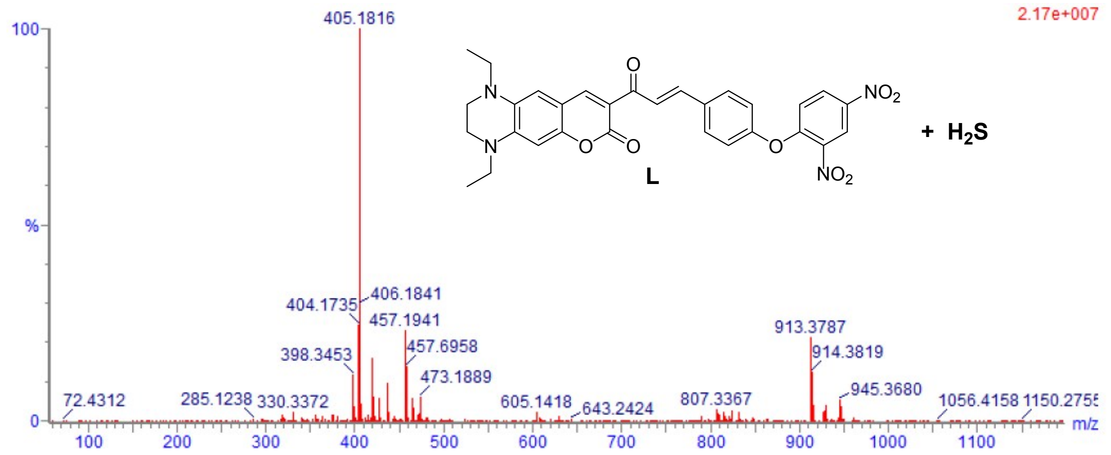

Figure S9. HRMS of compound **1** (above) and the reaction mixture of **L** with **HS**$^-$ (below).

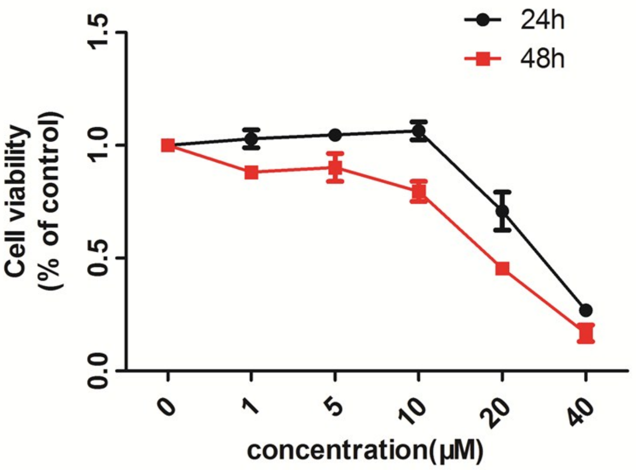

Figure S10. Cell viability values (%) estimated by MTT assay in MCF-7 cells, which were cultured in the presence of different concentrations of probe **L** (1.0, 5.0, 10, 20, and 40 μM).

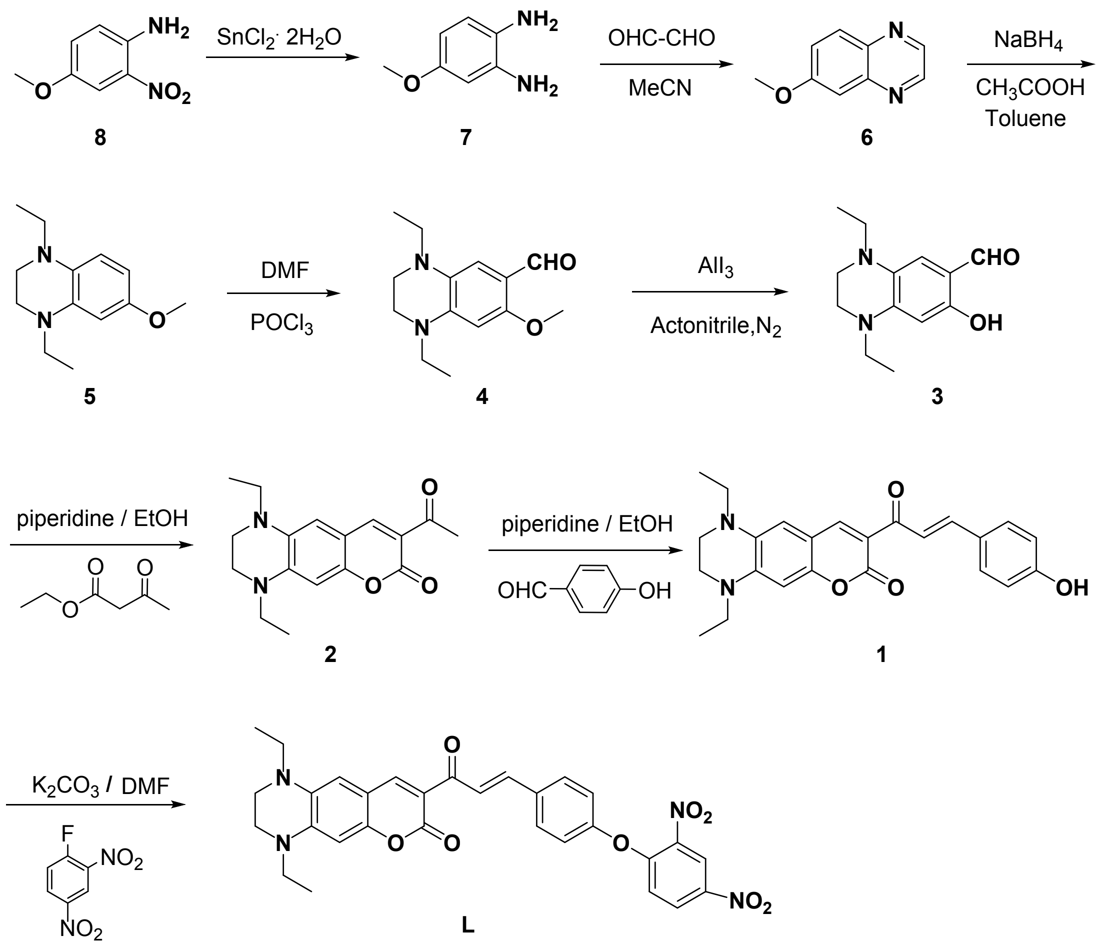

Scheme S1. Synthesis route of **L**.

Synthesis

The synthesis of compound 7

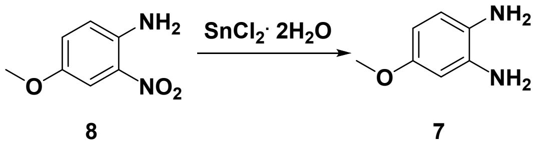

4-Methoxy-2-nitroaniline (20 g，0.12 mol), stannous chloride (72 g，0.6 mol) were dissolved in dry methanol (200 mL), then the mixture was refluxed for 12 h. After cooling, methanol was removed under reduced pressure. The mixture was washed with saturated $NaHCO_3$ and NaCl solution, and extracted with ethyl acetate. The organic layer was dried with anhydrous Na_2SO_4 and concentrated in rotavapour. The crude product **7** is oily liquid (15.2 g) and be used directly for next step without purification.

The synthesis of compound 6

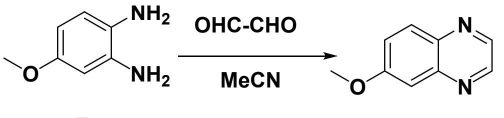

Compound **7** (15.2 g, 0.11 mol), glyoxal (40%, 49 mL, 4.0 mol) were added into in dry acetonitrile (100 mL), then the mixture was stirred at 60 °C for 12 h and cooled. The solvent was removed in a rotary evaporator and the crude product was purified by column chromatography with petroleum ether/ethyl acetate (10:1, v/v) as eluent to give white solid compound **6** (15.9 g, 89.7%). ^{1}H NMR (400 MHz, $CDCl_3$) δ 8.75 (d, *J* = 2.0 Hz, 1H), 8.68 (d, *J* = 2.0 Hz, 1H), 7.97 (d, *J* = 9.2 Hz, 1H), 7.41 (dd, *J* = 9.2, 2.8 Hz, 1H), 7.36 (d, *J* = 2.8 Hz, 1H), 3.96 (s, 3H).

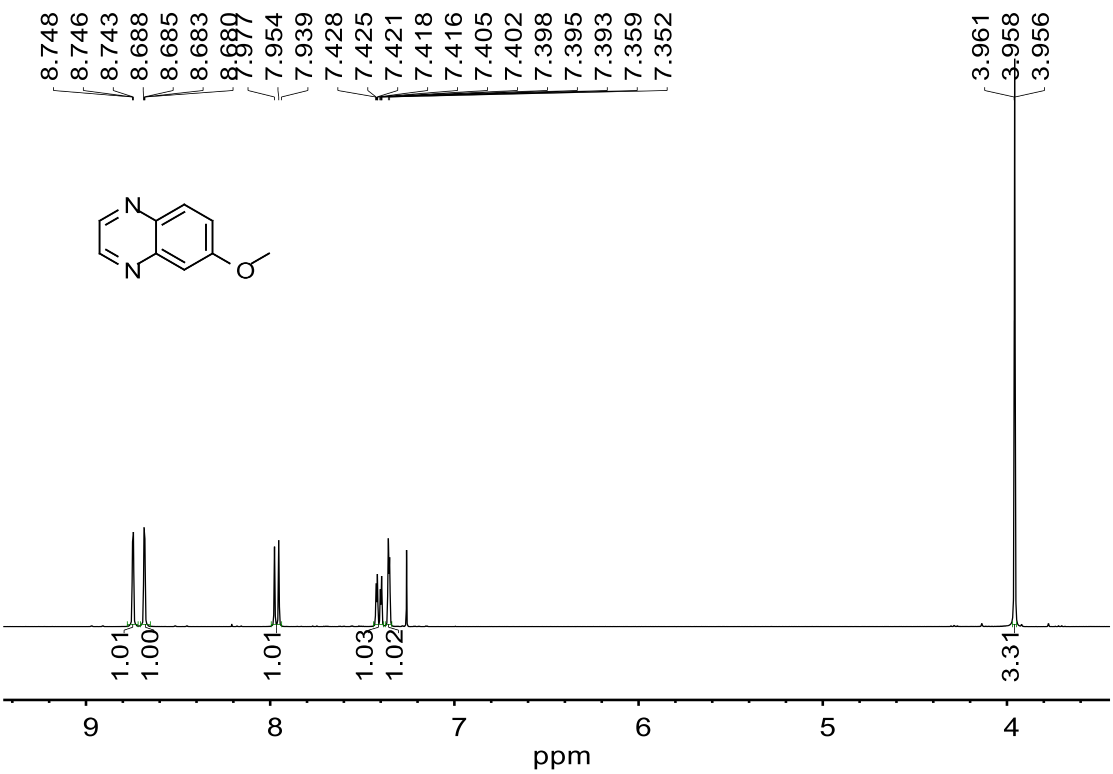

Figure S11. ^{1}H NMR spectrum of compound **6** in $CDCl_3$.

The synthesis of compound 5

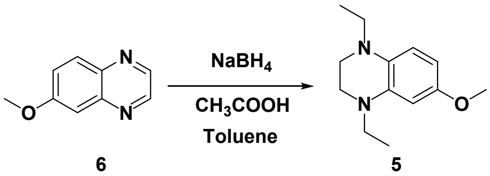

Compound **6** (15.9 g, 0.1 mol) was dissolved in dry toluene (200 mL), then sodium borohydride (38 g, 1.0 mol) was added into toluene over a period of 30 min at 0-5 ^{o}C. The obtained pale yellow slurry was stirred for 10 min, and then glacial acetic acid (57.3 mL, 60 g, 1.0 mol) was added dropwise over a period of 1 h at 5–10 ^{o}C. The brown slurry was stirred for another 1 h at 10 ^{o}C, and then heated to reflux for 5 h. After cooling, water (250 mL) was added, and the toluene layer was separated, then aqueous layer was extracted with ethyl acetate (3×100 mL). Combined extracts and toluene layer were washed repeatedly with dilute Na_2CO_3 and water, dried over anhydrous sodium sulphate, filtered and vacuum evaporated. The obtained dark brown oil was purified by column chromatography with petroleum ether/ethyl acetate (10:1, v/v) as eluent to give golden yellow oil compound **5** (13.4 g, 70%) ^{1}H NMR (400 MHz, $CDCl_3$) δ 6.57 (d, *J*=8.4 Hz, 1H), 6.24-6.29 (m, 2H), 3.81 (s, 3H), 3. 31 – 3.40 (m, 8H), 1.22 (t, J=7.2 Hz, 6H).

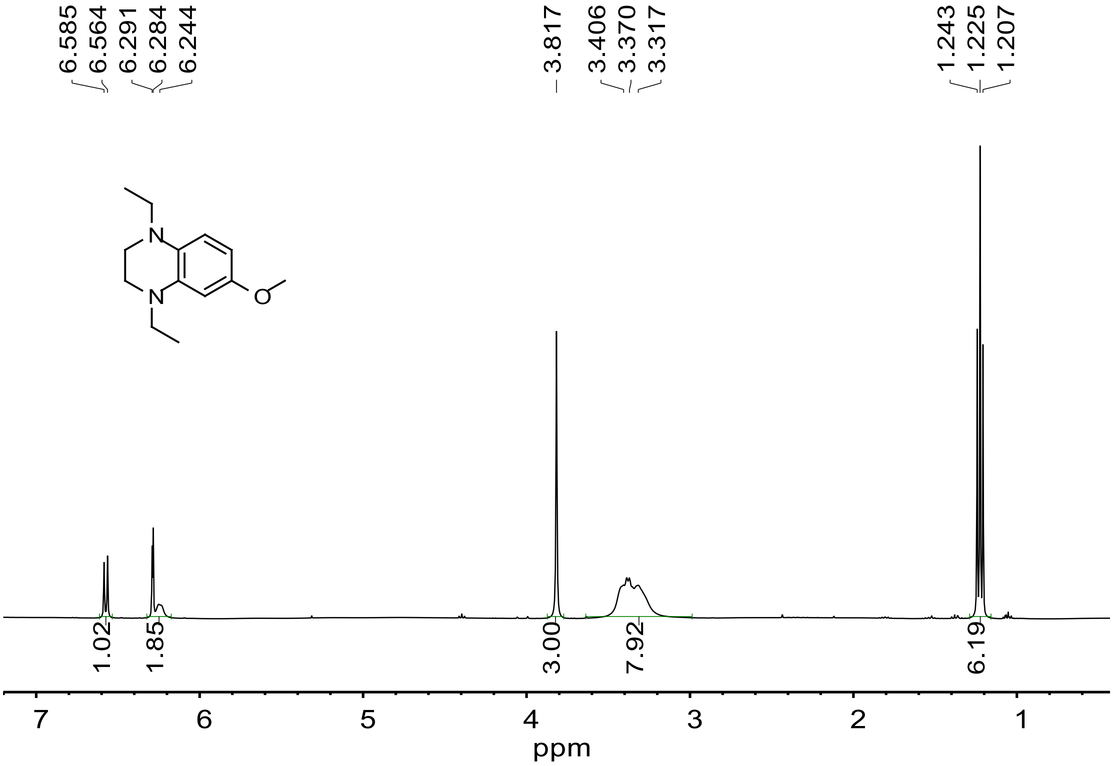

Figure S12. ^{1}H NMR spectrum of compound **5** in $CDCl_3$.

The synthesis of compound 4

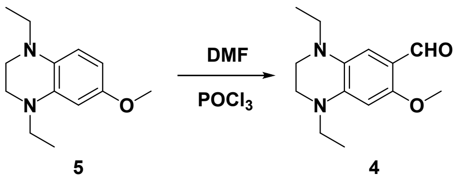

5 4

Phosphorus oxychloride (8.9 mL, 0.1 mol) was added into dimethyl formamide (DMF, 30 mL) under stirring at 0 °C. After 15 minutes, compound **5** (13.4 g，0.07 mol) diluted by 20 mL DMF was added into the cooled reagent with stirring. The mixture was heated at 75 °C for 6 h and then poured into icewater. The clear solution was neutralized by cold sodium hydroxide solution (15%) maintaining 10~15 °C. Combined organic layers were washed by water, dried over anhydrous sodium sulphate, filtered and vacuum evaporated. The brown sticky crude products were purified by column chromatography with petroleum ether/ethyl acetate (3:1, v/v) as eluent to give compound **4** (10.8 g, 78%). ^{1}H NMR (400 MHz, $CDCl_3$) δ 10.09 (s, 1H), 6.94 (s, 1H), 5.98 (s, 1H), 3.80 (s, 3H), 3.43-3.50(m, 2H), 3.37 (q, J = 7.1 Hz, 2H), 3.26 (q, J = 7.1 Hz, 2H), 3.07- 3.14 (m, 2H), 1.18 (t, J = 7.1 Hz, 3H), 1.11 (t, J = 7.1 Hz, 3H).

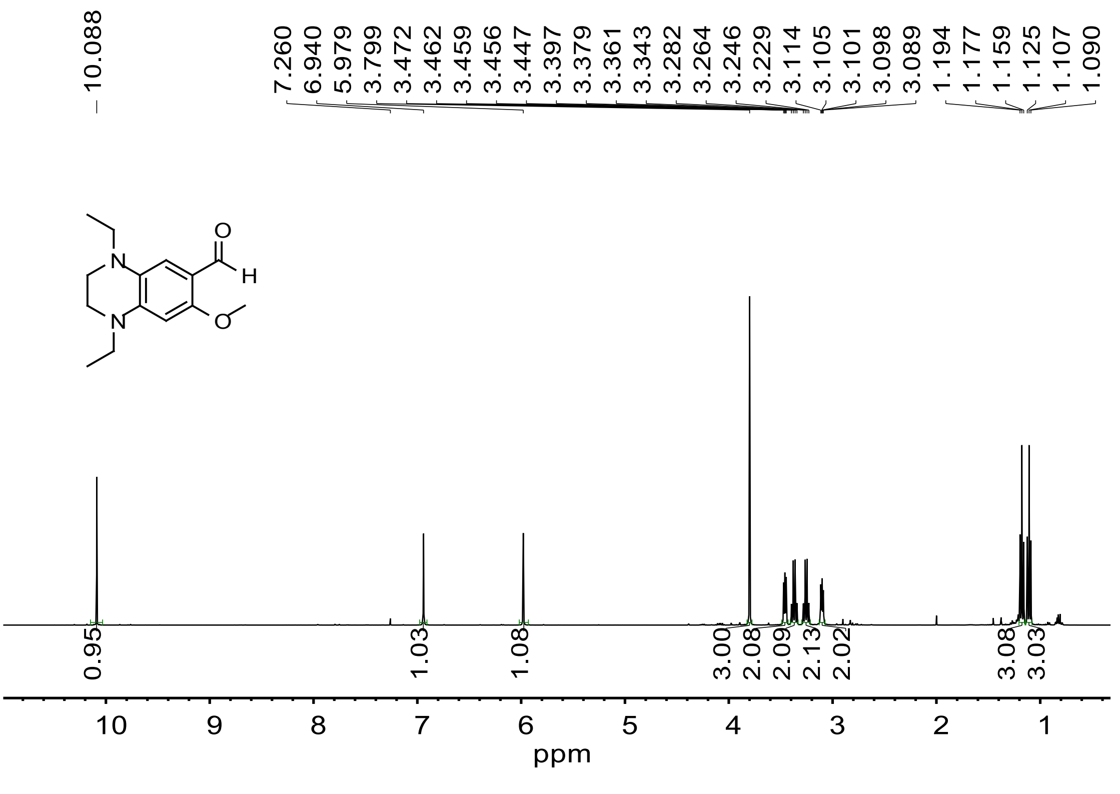

Figure S13. ^{1}H NMR spectrum of compound **4** in $CDCl_3$.

The synthesis of compound 3

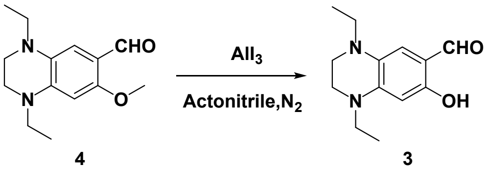

4 3

Aluminum powder (1.68 g, 0.06 mol) was dissolved in 30 mL acetonitrile, iodine (18.0 g, 0.14 mol) was added into the slurry by small portions and stirred under nitrogen atmosphere untill the colour changed to yellow. Compound **4** (10.8 g, 0.05 mol) diluted by 5 mL MeCN was added to the slurry dropwise. The mixture was then gently refluxed for 10 h, cooling and slowly poured into cold water (100 mL). The mixture was extracted with ethyl acetate (4×100 mL). Combined organic layers were washed by water, dried over anhydrous Na_2SO_4 and vacuum evaporated. The pale yellow oil products were purified by column chromatography with dichloromethane/ petroleum ether (1:1, v/v) as eluent to give compound **3** (7.7 g, 76%) ^{1}H NMR (400 MHz, DMSO-d_6) δ 10.97 (s, 1H), 9.55 (s, 1H), 6.59 (s, 1H), 5.96 (s, 1H), 3.44 (t, 2H), 3.33 (q, J = 7.0 Hz, 2H), 3.18 (q, J = 7.0 Hz, 2H), 3.04 (t, 2H), 1.04 (t, J =7.2 Hz, 3H), 1.06 (t, J =7.2 Hz, 3H).

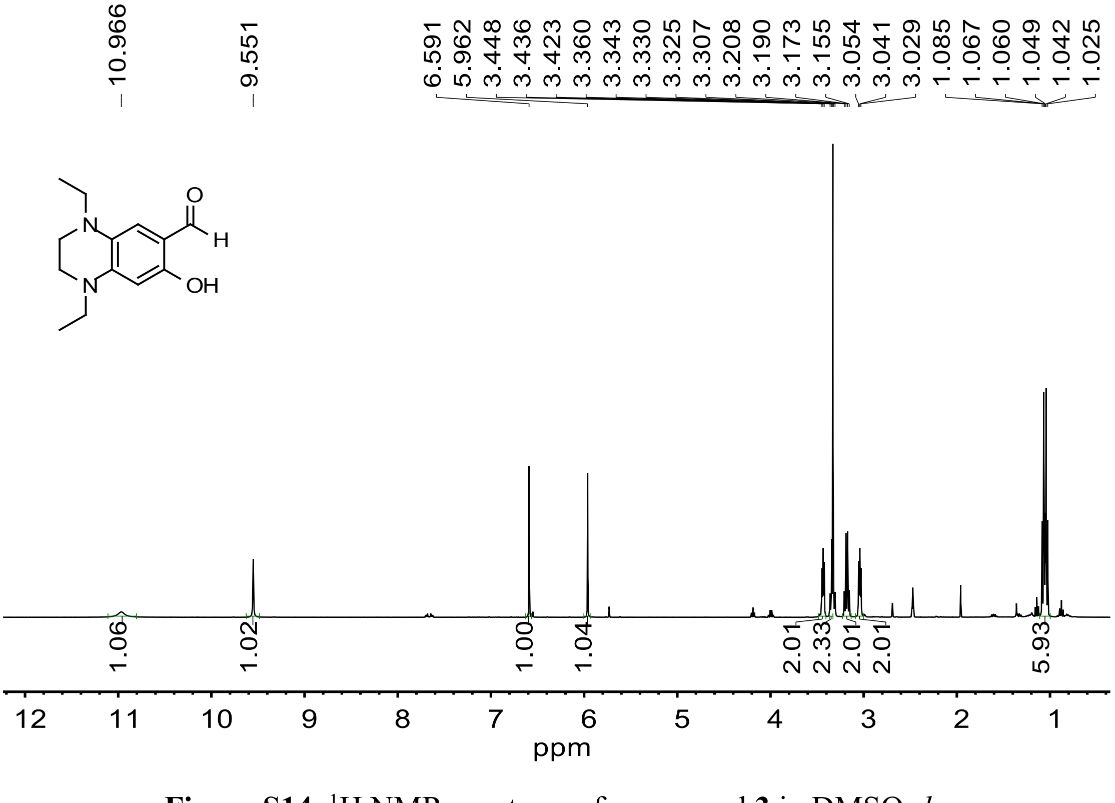

Figure S14. ^{1}H NMR spectrum of compound **3** in DMSO-d_6.

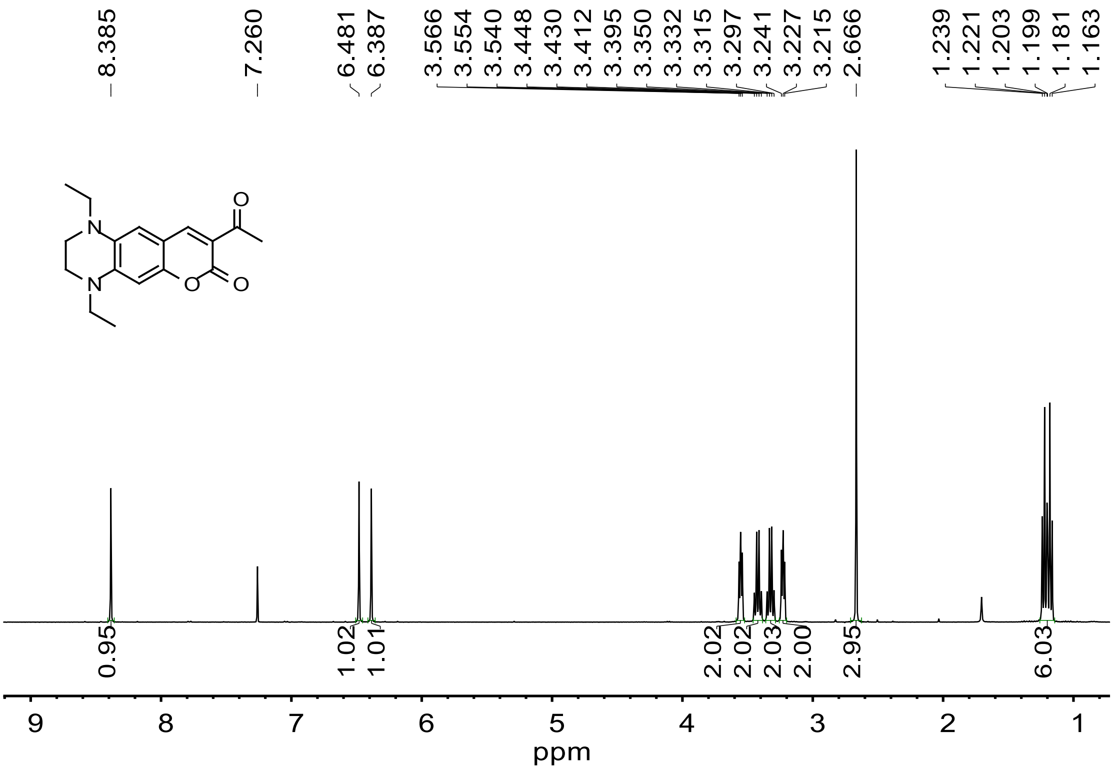

Figure S15. ^{1}H NMR spectrum of compound **2** in $CDCl_3$.

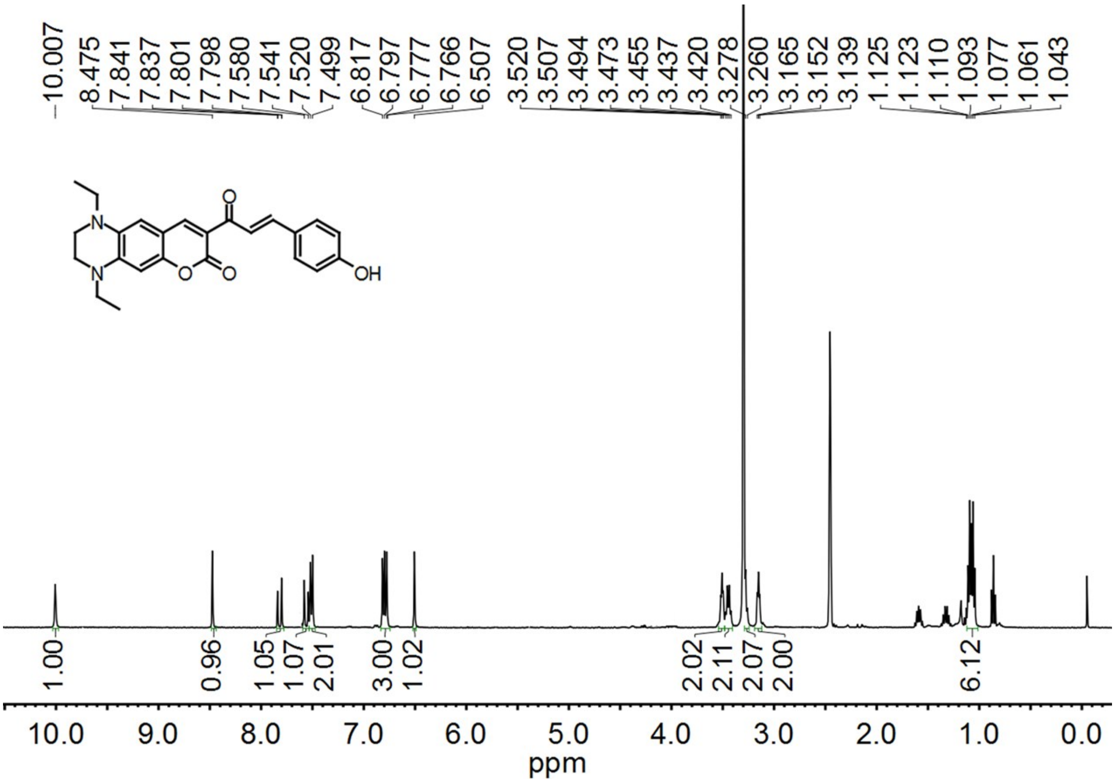

Figure S16. ^{1}H NMR spectrum of compound **1** in DMSO-d_6.

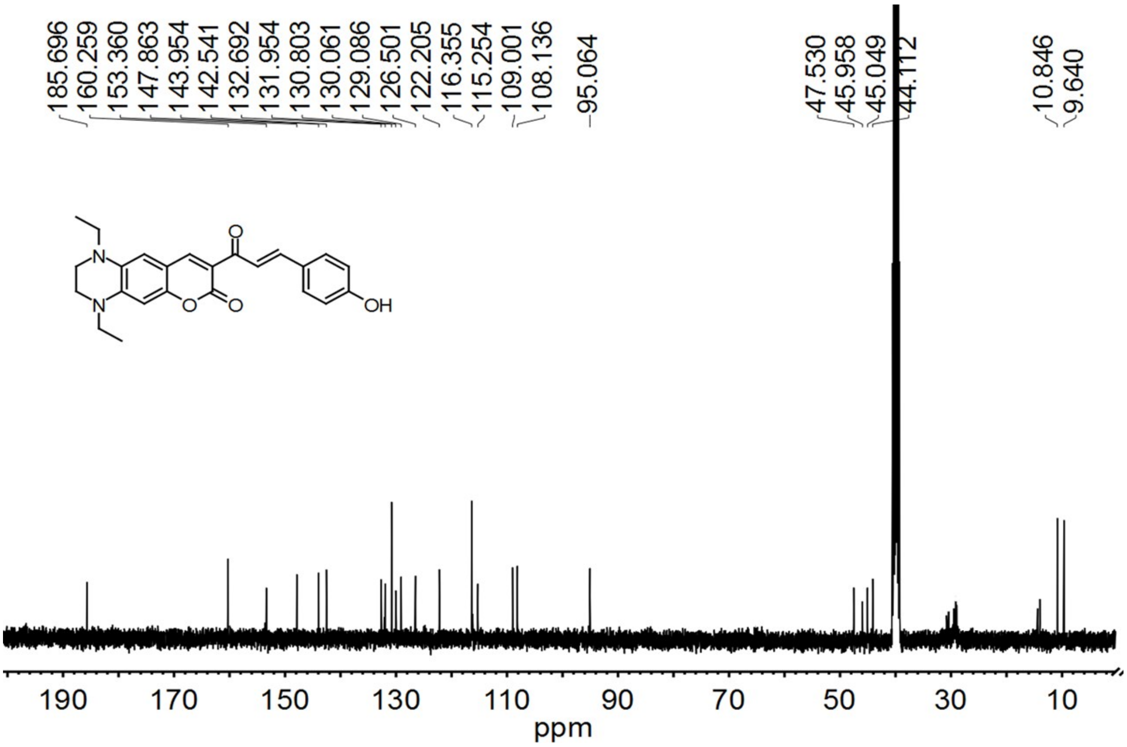

Figure S17. ^{13}C NMR spectrum of compound **1** in DMSO-d_6

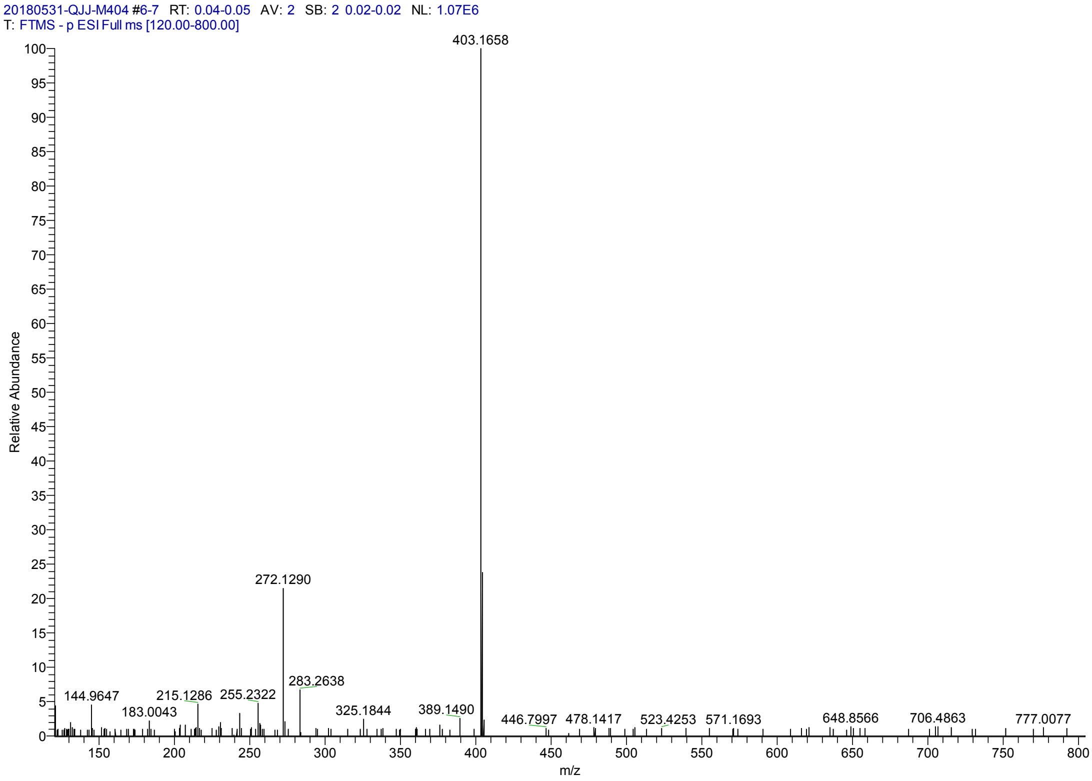

Figure S18. HRMS spectrum of compound **1** in CH_3CN ($[M-H]^+$ calcd: 403.1736, found: 403.1658) .
